# Knockdown of Long Noncoding RNA 01124 Inhibits the Malignant Behaviors of Colon Cancer Cells via miR-654-5p/HAX-1

**DOI:** 10.1155/2022/1092107

**Published:** 2022-09-20

**Authors:** Yu-jin Wu, Zhi-quan Cai, Run-ming He, Xue-chuan Wang, Long-ling Cong, Fang-hua Qiu

**Affiliations:** ^1^Departments of Gastroenterology, The Affiliated TCM Hospital of Guangzhou Medical University, Guangzhou, Guangdong 510130, China; ^2^Department of Hospital Office, The Affiliated TCM Hospital of Guangzhou Medical University, Guangzhou, Guangdong 510130, China

## Abstract

**Background:**

Previous studies have shown that long noncoding RNAs (lncRNAs) play a key role in cancer, including colon cancer (CC). However, the exact role of long noncoding RNA 01124 (LINC01124) in CC and its mechanisms of action remain unknown. In this study, we investigated the functional effects and the possible mechanism of LINC01124 in CC.

**Methods:**

We first determined the expression of LINC01124 in CC tissues (The Cancer Genome Atlas (TCGA) database) and cell lines (quantitative real-time polymerase chain reaction (qRT-PCR)). Functional analysis via Cell Counting Kit-8 (CCK-8), colony formation, cell cycle, wound healing and Transwell assays were performed, and a mechanistic experiment was performed with the western blotting. The function of LINC01124 was also determined in vivo using nude BALB/c mice.

**Results:**

The results showed that LINC01124 was upregulated in CC tissues and cell lines. Functional studies showed that knockdown of LINC01124 significantly suppressed the proliferation, migration, and invasion of colon cancer cells in vitro and in vivo. Subsequent mechanistic experiments indicated that LINC01124 acted as a sponge to suppress microRNA 654-5p, which targeted HAX-1. Downregulation of LINC01124 decreased the expression of HAX-1, and overexpression of the miR-654-5p inhibitor attenuated the sh-LINC01124-induced inhibition of CC cell proliferation, migration, and invasion.

**Conclusion:**

Collectively, this study revealed that the knockdown of LINC01124 inhibited the malignant behaviors of CC via the miR-654-5p/HAX-1 axis, suggesting that LINC01124 might be a therapeutic target for CC treatment.

## 1. Background

Colon cancer (CC) is the third most common malignancy worldwide [1], with more than 1.2 million newly diagnosed patients resulting in 551 thousand deaths each year worldwide [2]. High morbidity and mortality make colon tumors a serious threat to human health [3]. The lack of early diagnosis is one of the most important reasons for the high incidence of CC [4]. Another important reason is that malignant CC often possesses the characteristics of rapid progression and invasion, which can result in a poor prognosis [5, 6]. Therefore, identifying key molecules in the development of colorectal cancer (CRC) tumors may provide breakthroughs in the targeting and biomarker selection of antitumor drugs for this malignant disease.

Long noncoding RNA (lncRNA) is a nonprotein coding RNA transcript with a length greater than 200 nucleotides (nt) [7]. lncRNAs were found to be involved in regulating various processes, and their dysfunctions have been associated with the occurrence of many diseases, including tumors [8]. They can exert their effects on gene expression at transcriptional and post-transcriptional levels [9, 10]. For instance, lncRNAs can bind to DNA, RNA, and proteins to influence transcriptional initiation, RNA stability, or the activity of signaling pathways [10]. They can also serve as scaffolds for recruiting transcriptional factors to the promoter region to affect gene expression. The involvement of lncRNAs and their critical roles have been reported in the development of cancers, such as malignant proliferation, metastasis, invasion, antiapoptosis effects, and therapeutic resistance [8, 11]. The dysregulation of many lncRNAs, such as lncRNA CCAT1, lncRNA POU6F2-AS2, and lncRNA ROR1-AS1, has been closely related to CRC progression [12, 13]. A previous study also confirmed that LINC01124 significantly inhibited the proliferation, migration, and invasive ability of non-small-cell lung carcinoma (NSCLC) cells [14]. However, the potential effect and mechanism of LINC01124 on CC remain unclear. Therefore, we decided to explore the potential functions of LINC01124 in CC.

HAX-1, located at chromosome 1 (1q21.3), is reported to play an important role in various tumors [15–18]. For example, HAX-1 was revealed to be overexpressed in hypopharyngeal squamous cell carcinoma and promoted cancer growth and migration [17]. HAX-1 was found to be targeted by miR-100 to regulate the sensitivity of breast cancer cells to cisplatin [16]. Moreover, in CRC, HAX-1 was reported to be targeted by miR-654-5p to regulate its malignancy behaviors [18].

LINC01124 is located at chromosome 2q31.1 and contains one exon. In the current study, we investigated the role of LINC01124 in the progression of CC and explored its underlying mechanisms. The results showed that LINC01124 was upregulated in CC tissues and cell lines. Knockdown of LINC01124 suppressed cell proliferation, migration, and invasion in vitro and in vivo. In addition, miR-654-5p was verified to be target miRNA of LINC01124, and we showed that sh-LINC01124 inhibited the progression of CC by modulating the miR-654-5p-HAX-1 signaling pathway. All these findings suggested that LINC01124 might be an underlying biomarker and a potential therapeutic target for CC.

## 2. Materials and Methods

### 2.1. Cell Culture

A human normal colonic epithelial cell line (NCM460) and 5 human CC cell lines (LoVo, SW620, HT29, HCT116, and SW480) were purchased from Nanjing Cobioer Biotechnology Co. Ltd. (Nanjing, Jiangsu, China). All CRC cell lines were cultured in RPMI 1640 (Roswell Park Memorial Institute 1640) medium supplemented with 10% fetal bovine serum (FBS, Gibco, Grand Island, NY, USA) and 1% penicillin-streptomycin. All cells were maintained in an incubator at 37°C with 5% CO_2_ in a humidified atmosphere [19].

### 2.2. Transfection and Lentivirus Transduction

The miR-654-5p inhibitor and its negative control (Ctrl) were purchased from RiboBio, and oligonucleotide transfection was performed using Lipofectamine 2000 reagent (Invitrogen; Thermo Fisher Scientific, Inc.). Short hairpin RNA (shRNA) targeting LINC01124 was designed by GenePharma (Shanghai, China) and cloned into the pRNAT-u6.1/Neo plasmid (Biovector, Beijing, China). To establish a cell line with stable knockdown of LINC01124, the plasmid carrying sh-LINC01124 or sh-Ctrl was cotransfected with packaging vectors to produce pseudotyped lentiviruses, which were designated Lv-sh-LINC01124 and Lv-sh-Ctrl. The lentiviruses were concentrated by ultracentrifugation and then were used to infect CC cells [19].

### 2.3. Quantitative Real-Time Polymerase Chain Reaction (qRT-PCR)

After transfection, total RNA was extracted from cells by TRIzol extraction (Invitrogen; Thermo Fisher Scientific, Inc.). Then, RNA samples were reverse transcribed into complementary DNA (cDNA) using a PrimeScript RT reagent kit (Takara). qRT-PCR analyses were performed with SYBR Green (Takara). The primers are shown in Table 1. The results were normalized to the expression of glyceraldehyde-3-phosphate dehydrogenase (GAPDH). Relative gene expression levels were calculated using the 2^−ΔΔct^ method [14].

### 2.4. Cell Proliferation Analysis

For the Cell Counting Kit-8 (CCK-8) assay, 2000 cells were seeded into 96-well plates in 100 *μ*l of complete medium and cultured for 1, 2, 3, 4, and 5 days. At each time point, 10 *μ*L of the CCK‐8 solution (Dojindo, Kumamoto, Japan) was added to each well, and the absorbance was then measured at 450 nm using a microplate reader after 2 h at 37°C. The cells' cell viability was determined by measuring their absorbance. For colony formation assays, the cells were seeded into 12-well plates at a density of 500/well and incubated for 10–12 days. Then, colonies were stained with 0.1% crystal violet (Sigma) and counted [20].

### 2.5. Cell Cycle Analysis

HCT116 and SW480 cells were washed with cold PBS and then fixed with ice-cold 70% ethanol at 4°C overnight. On the second day, the cells were washed with PBS, and intracellular DNA was labeled with propidium iodide (PI, Sigma-Aldrich; Merck KGaA) at 4°C for 30 min and analyzed using BD FACSCalibur flow cytometry (BD Bioscience, San Jose, CA, USA). ModFit software (Verity Software House Inc., Topsham, ME, USA) was used to analyze the proportions of cells in the GO/G1, S, and G2/M phases [19].

### 2.6. Wound Healing Assay

Cells (1 × 10^5/well) were seeded into 6-well plates. The cells were starved in fetal bovine serum (FBS) free culture medium overnight. Then, a wound was made using a 200 *μ*l pipette tip. Next, the cells were incubated with 2% FBS medium. The wound was imaged at 0 h and 36 h [20].

### 2.7. Transwell Invasion Assay

The transwell chamber (8-*μ*m pore size, Corning, Cambridge, MA, USA) was used to perform the invasion assay. Cells (2 × 105/well) were cultured in the upper chamber with Matrigel (BD Biosciences), and a complete medium containing 20% FBS was added to the lower chamber. After incubation for 36 h at 37°C, cells adhering to the lower surface of the transwell membrane were fixed in 20% methanol and stained with 0.1% crystal violet. The number of invaded cells was analyzed [20].

### 2.8. Tumorigenesis Assay In Vivo

For the subcutaneous xenograft assay, 5 × 105 cells infected with sh-Ctrl or sh-LINC01124 were subcutaneously inoculated into the flanks of 5-week-old male athymic nude BALB/c mice. The tumor volumes were examined using calipers every three days. After 5 weeks, the mice were sacrificed by euthanasia. The tumors were then removed and weighed [19]. All animal studies were performed in strict accordance with the recommendations in the guidelines for the Animal Care and Use Committee of the Traditional Chinese Medicine University of Guangzhou (Permit number: 20190228035).

### 2.9. Luciferase Reporter Assay

The binding sequences of miR-654-5p and LINC01124 were predicted through online websites (https://www.mircode.org/and https://cm.jefferson.edu/ran22/Interacti-ve/). The potential binding sequences of miR-654-5p and LINC01124 (WT) and the mutant (Mut) miR-654-5p binding sequences in LINC01124 were inserted into a pmirGL3-basic vector (Promega Corporation, Madison, USA) to construct dual-luciferase reporter plasmids. Then, the cells were cotransfected with the WT (or Mut) plasmid and a Ctrl mimic or miR-654-5p mimic. After 48 h, luciferase activity was detected using a dual-luciferase reporter gene assay kit (Beyotime Institute of Biotechnology, Shanghai, China) according to the manufacturer's protocol. The relative luciferase activity was normalized to Renilla luciferase activity [20].

### 2.10. Western Blot

Protein samples were isolated with lysis buffer (RIPA) containing protease inhibitors. After quantification with a BCA kit (Beyotime, China), total protein (25 *μ*g) was separated by 8–15% SDS-PAGE and transferred to polyvinylidene difluoride (PVDF) membranes. After blocking with 5% skimmed milk for 60 min, the membranes were incubated with primary antibodies anti-HAX-1 (ab137613, 1 : 500) and anti-GAPDH (#5174, CST, 1 : 1000) overnight, followed by incubation with fluorescence-conjugated secondary antibodies (1 : 1,000) for 30 min. Bands were detected by using a two-color infrared laser imaging system (Odyssey; Li-Cor, Lincoln, NE, USA) [20].

### 2.11. Statistical Analysis

All data in this study were obtained from experiments repeated at least three times and are presented as the mean ± standard deviation (SD). Two independent sample *t*-tests or one-way ANOVA for multiple comparisons were performed using SPSS v22.0 (IBM, Armonk, NY, USA). ^*∗∗*^*p* < 0.01 or ^*∗*^*p* < 0.05 was considered statistically significant [20].

## 3. Results

### 3.1. The Expression of LINC01124 Was Upregulated in CC Tissues and Cell Lines

**Figure 1:**
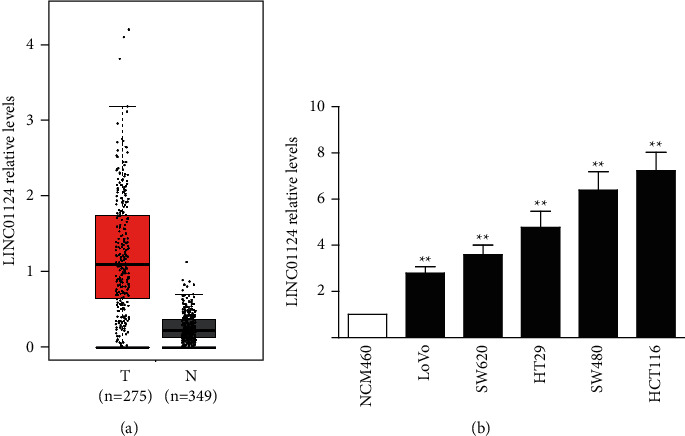
LINC01124 was upregulated in human colon cancer tissues and cell lines. (a) LINC01124 expression was analyzed in online public databases comparing 275 colon cancer tissues and 349 adjacent normal tissues. (b) Comparison of the relative LINC01124 expression levels between the normal colonic epithelial cell line (NCM460) and 5 CRC cell lines. Data are presented as the mean ± SD, ^*∗∗*^*p* < 0.01.

To investigate the role of LINC01124 in CRC, we first analyzed the expression levels of LINC01124 in The Cancer Genome Atlas (TCGA) database (https://gepia.cancer-pku.cn/index.html). As shown in Figure 1(a), the expression of LINC01124 was significantly upregulated in CRC tissues. Next, we analyzed the expression of LINC01124 in CC cell lines. As illustrated in Figure 1(b), the expression of LINC01124 in all CC cell lines (LoVo, SW620, HT29, HCT116, and SW480) was significantly higher than that in the normal colonic epithelial cell line (NCM460). HCT116 and SW480 cells were chosen for downstream experiments based on their high expression. Our results show that LINC01124 was overexpressed in CC cell lines.

### 3.2. LINC01124 Knockdown Inhibited Cell Proliferation In Vitro

**Figure 2:**
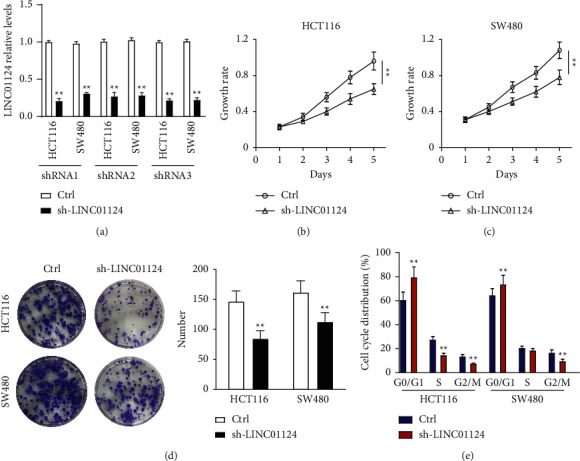
Knockdown of LINC01124 inhibited cell proliferation in vitro. (a) The inhibitory efficiency of sh-LINC01124 in LINC01124 expression by qRT-PCR. (b, c) CCK-8 assays and (d) colony formation assays were performed to assess the proliferation of HCT116 and SW480 cell lines. (e) Flow cytometry analysis was used for cell cycle evaluation. Data are presented as the mean ± SD, ^*∗∗*^*p* < 0.01.

Loss-of-function experiments were performed by knocking down LINC01124 to explore the regulatory effect of LINC01124 on CC cell progression. We knocked down LINC01124 in HCT116 and SW480 cells. qRT-PCR results showed that the expression of LINC01124 was significantly downregulated in these cells by transfecting sh-LINC01124 (Figure 2(a)). We then performed CCK-8 and colony formation assays to evaluate cellular proliferation. The results demonstrated that knockdown of LINC01124 significantly inhibited cell proliferation (Figures 2(b)–2(d) and Supplementary Figure 1). More importantly, FACS analysis indicated that after transfection with sh-LINC01124, fewer cells entered the S phase and G2/M phase, while more cells were arrested in the G0/G1 phase (Figure 2(e)). Collectively, these findings suggest that knockdown of LINC01124 inhibited CC cell proliferation in vitro.

### 3.3. LINC01124 Knockdown Reduced Tumor Growth In Vivo

**Figure 3:**
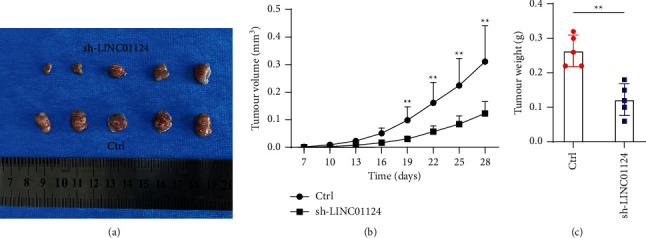
Knockdown of LINC01124 reduced tumor formation in nude mice xenografts. (a) Representative photos of xenografts. (b) The volumes in subcutaneous xenografts measured and calculated once a week for 5 weeks. (c) The tumor weight measured at the end of the experiments. Data are presented as the mean ± SD, ^*∗∗*^*p* < 0.01.

We further investigated the function of LINC01124 in vivo. sh-LINC01124 or control SW480 cells were subcutaneously injected into the flanks of nude mice. From the first week, we measured the tumor volumes at indicative time points. After 5 weeks, the mice were sacrificed, and the tumors were removed. As shown in Figures 3(a) and 3(b), knockdown of LINC01124 significantly inhibited tumor growth in vivo. Moreover, the tumors in the sh-LINC01124 group were smaller than those in the control group (Figure 3(c)). These findings indicate that knockdown of LINC01124 could reduce CC progression in vivo.

### 3.4. LINC01124 Knockdown Inhibited Cell Migration and Invasion

**Figure 4:**
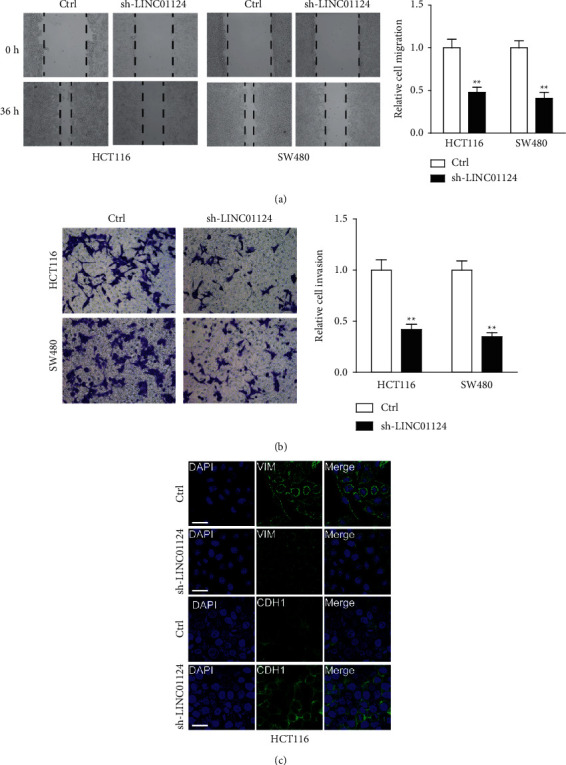
Knockdown of LINC01124 inhibited cell migration and invasion. (a) Wound healing assay to evaluate the effect of LINC01124 on the migration of HCT116 and SW480 cells (magnification, ×100). (b) Transwell assay to evaluate the effect of LINC01124 on the invasion of HCT116 and SW480 cells (magnification, ×200). (c) HCT116 cells were harvested for IF analysis by anti-VIM or anti-CHD1 (scale bar, 15 *μ*m). Data are presented as the mean ± SD, ^*∗∗*^*p* < 0.01.

We assessed the effects of LINC01124 depletion on cell migration and invasion using wound healing and transwell assays. As expected, knockdown of LINC01124 significantly suppressed the migration and invasion of HCT116 and SW480 cells (Figures 4(a) and 4(b)). Moreover, the expression of metastasis-related protein VIM was significantly downregulated, while another metastasis-related protein, CDH1, was upregulated in HCT116 cells transfected with sh-LINC01124 (Figure 4(c)). Collectively, these findings indicate that LINC01124 may act as an oncogene in CC.

### 3.5. LINC01124 Knockdown Inhibited the Progression of CC via the miR-654-5p/HAX-1 Axis

**Figure 5:**
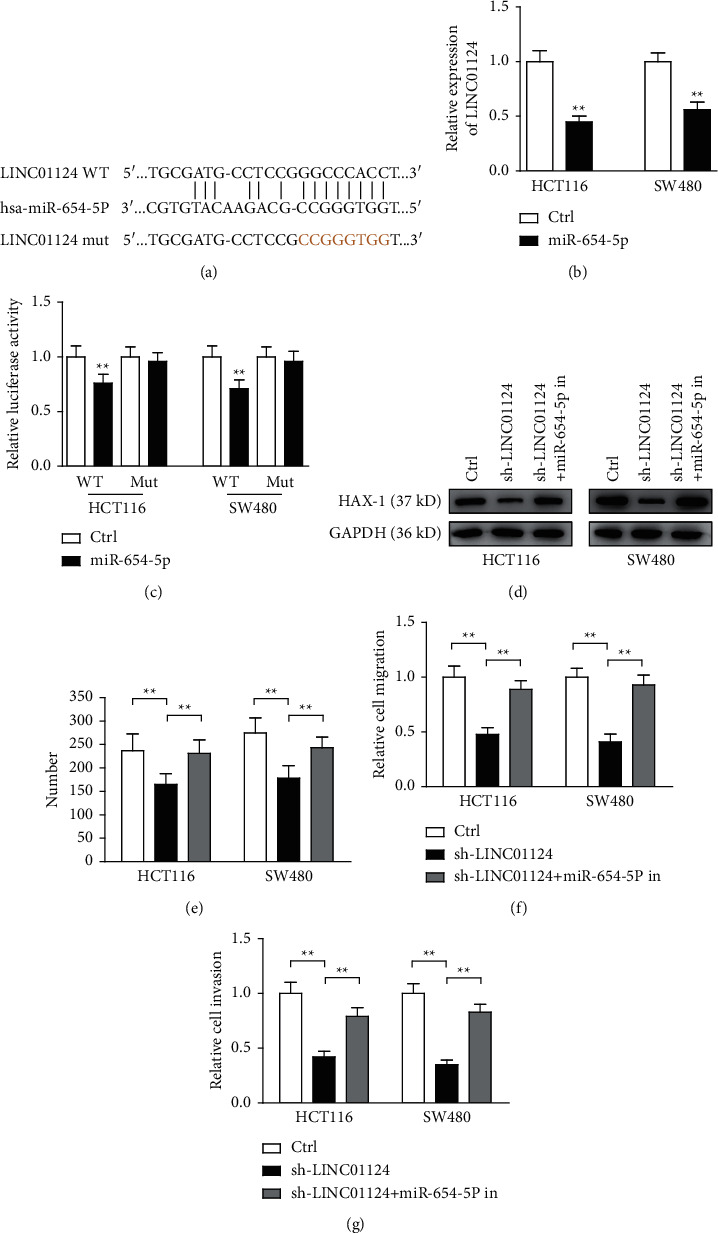
Knockdown of LINC01124 inhibited the progression of CC via the miR-654-5p/HAX-1 axis. (a) Predicted binding sites in LINC01124 of miR-654-5p. (b) Overexpression of miR-654-5p reduced the expression of LINC01124 in HCT116 and SW480 cell lines. (c) A dual-luciferase reporter assay was performed to assess the luciferase activity of HCT116 and SW480 cell lines transfected with WT-LINC01124 and mut-LINC01124. (d) Western blotting analysis of HAX-1 in HCT116 and SW480 cell lines. (e–g) Colony formation, wound healing, and transwell assays were performed with the indicated cell lines. Data are presented as the mean ± SD, ^*∗∗*^*p* < 0.01.

lncRNAs have been demonstrated to act as competing endogenous RNAs for miRNAs. Thus, two different mRNA target prediction algorithms, miRcode and RNA22, were used to predict the potential miRNAs that directly bind to LINC01124. Among all potential targets, we identified miR-654-5p, whose expression was downregulated in CC cells [18], as the most promising candidate. The potential binding sequences of miR-654-5p and LINC01124 are shown in Figure 5(a). Overexpression of miR-654-5p significantly suppressed the expression of LINC01124 (Figure 5(b)), and overexpression of LINC01124 significantly suppressed the expression of miR-654-5p (Supplementary Figure 2). To confirm the association of LINC01124 and miR-654-5p, the expression levels of these two RNAs were analyzed using TCGA data. As shown in Supplementary Tables 1, 2 and Supplementary Figure 3, there was an obvious negative correlation between LINC01124 and miR-654-5p, *r* = 0.556966. Moreover, the luciferase reporter assay indicated that overexpression of miR-654-5p suppressed the luciferase activity in HCT116 and SW480 cells transfected with the WT-LINC01124 vector (Figure 5(c)). It has been reported that miR-654-5p targets HAX-1 to regulate the malignant behaviors of CRC cells [18]; thus, we detected the expression of HAX-1 by the western blotting in HCT116 and SW480 cell lines transfected with sh-LINC01124 or cotransfected with sh-LINC01124 and miR-654-5p inhibitors. As shown in Figure 5(d), the expression of HAX-1 decreased when LINC01124 was knocked down; however, the opposite expression pattern of HAX-1 was observed when sh-LINC01124 and miR-654-5p inhibitors were cotransfected. Altogether, these findings demonstrated that LINC01124 is directly bounded to miR-654-5p in CC.

After confirming the direct interaction between miR-654-5p and LINC01124, rescue experiments were performed. sh-LINC01124 and miR-654-5p inhibitors were cotransfected into both HCT116 and SW480 cell lines. By assessing cell proliferation, migration, and invasion using colony formation, wound healing and transwell assays, we found that the miR-654-5p inhibitor partly reversed the suppressive effect of sh-LINC01124 on cell proliferation, migration, and invasion (Figures 5(e)–5(g)). Taken together, our findings suggested that LINC01124 acted as an oncogene via inhibition of miR-654-5p by targeting HAX-1.

## 4. Discussion

Due to limitations in the early diagnosis of CC, its 5-year survival rate is still less than 30% in many low-income countries [21, 22]. An increasing number of methods are being used to explore the pathogenesis of cancers, and accumulating evidence suggests that lncRNAs have important biological functions [23–25]. lncRNAs were once considered to be “transcriptional noise,” but now lncRNAs have been proven to be involved in the regulation of tumorigenesis and progression as tumor suppressor genes or oncogenes [26–28].

For the past few years, an increasing number of researchers have focused on the effect of lncRNAs on CC. For example, some scholars have shown that lncRNA ROR1-AS1 promoted the proliferation of CC by suppressing the expression of DUSP5/CDKN1A [12], lncRNA NEAT1 regulated invasion and migration in CC via the miR-185-5p/IGF2 axis [29], lncRNA LINC00460 knockdown suppressed EMT in CC by downregulating ANXA2 [30], lncRNA POU6F2-AS2 promoted drug resistance in CC by regulating miR-377/BRD4 [13], and more. LINC01124, which is located at chromosome 2q31.1, was shown to act as a tumor suppressor in NSCLC. The expression level of LINC01124 was reported to be downregulated in tumor tissues compared with paired normal lung tissues, and LINC01124 significantly inhibited the proliferation, migration, and invasive ability of NSCLC cells [14]. However, the role of LINC01124 in CC remained unclear. Here, we report for the first time that LINC01124 is upregulated in CC tissues and cell lines. The results showed that the downregulation of LINC01124 suppressed the proliferation, migration, and invasion of CC cell lines in vitro and in vivo. This function of LINC01124 in CC was the opposite of NSCLC cells.

Many studies have revealed some potential biological mechanisms of lncRNAs, and the theory of competing for endogenous RNAs (ceRNAs) is one of the commonly accepted theories [31]. ceRNAs can directly bind to miRNAs and affect the expression of target mRNAs, thus affecting the biological process of cells [32–34]. LINC01124 has not been reported to function as a ceRNA until recently, so we hypothesized that LINC01124 could regulate CC progression by binding to miRNAs. Using an online database, we found that miR-654-5p had a higher score for binding to LINC01124 and suppressed CC cell proliferation, migration, and invasion [18]. The luciferase reporter assay confirmed our prediction that LINC01124 could bind to miR-654-5p directly.

Hematopoietic cell-specific protein 1 (HS-1)-associated protein X-1 (HAX-1) is a 35 kDa protein that can interact with HS-1, an Src kinase substrate. HAX-1 was reported to be composed of a putative transmembrane domain, a putative PEST sequence, and an acid box [35]. It has emerged as an important factor in the mitochondrial-dependent cell death pathway, characterized by the activation and permeabilization of mitochondria, resulting in the release of cytochrome c and other proapoptotic molecules into cytosol [36]. Previous studies demonstrated that HAX-1 was involved in regulating mitochondrial membrane potential during apoptosis [37, 38]. In addition, HAX-1 protein was shown to interact with several cellular and viral proteins [39, 40]. Despite several studies demonstrating that HAX-1 might be important in apoptosis and proliferation [41, 42], its role in CRC remains under-investigated. In this present study, our experiments showed that the downregulation of LINC01124 significantly decreased the levels of HAX-1, which is the target gene of miR-654-5p. In addition, the miR-654-5p inhibitor rescued the effects of sh-LINC01124 on CC cell proliferation, migration, and invasion. All these findings suggested that the miR-654-5p/HAX-1 axis might be involved in the anti-CC effect of sh-LINC01124. Further bioinformatics analyses and in vitro and in vivo studies are needed to further validate the potential clinical significance of this axis in CC.

There were several limitations in this study. First, we did not use CC patients' tissues to determine the expression of LINC01124, which was only determined in the TCGA database. Second, immunohistochemistry was not performed to determine the association of LINC01124 with the survival of CC patients. Third, drug experiments were not performed to identify the optimal pharmacological therapies for patients overexpressing LINC01124. These issues could be clarified in future studies to confirm the clinical and real-world significance of LINC01124 as a therapeutic target for CC.

Taken together, our data for the first time showed that LINC01124 was upregulated in CC tissues and cell lines. Downregulation of LINC01124 was involved in malignant behaviors in CC. In addition, we identified miR-654-5p as a target of LINC01124. Thus, these findings indicate the potential role of the LINC01124/miR-654-5p/HAX-1 axis in CC, suggesting LINC01124 as a novel diagnostic and therapeutic target for CC.

## Figures and Tables

**Table 1:** Sequences of primers for qRT-PCR.

Name	Sequence (5' to 3')
PCR primers for GAPDH	
Forward	GTGGCTGGCTCAGAAAAAGG
Reverse	GGGGAGATTCAGTGTGGTGG

PCR primers for LINC01124	
Forward	GGACTCCGAGCTTTCAACCA
Reverse	AGCGATCTGGTTCCTTAGCG

## Data Availability

The datasets used and/or analyzed during the current study are available from the corresponding author on reasonable request.

## Supplementary Materials

Supplementary Materials Table S1: analysis of the expression levels of two RNAs LINC01124 and miR-654-5p using TCGA data. Table S2: analysis of the expression levels of two RNAs LINC01124 and miR-654-5p using TCGA data. Figure S1: knockdown of LINC01124 inhibited cell proliferation. Figure S2: the expression of miR-654-5p in ov-LINC01124 cells. Figure S3: the relationship of miR-654-5p and LINC01124.

## References

[1] Weitz J., Koch M., Debus J., Hohler T., Galle P. R., Buchler M. W. (2005). Colorectal cancer. *The Lancet*.

[2] Bray F., Ferlay J., Soerjomataram I., Siegel R. L., Torre L. A., Jemal A. (2018). Global cancer statistics 2018: GLOBOCAN estimates of incidence and mortality worldwide for 36 cancers in 185 countries. *CA: A Cancer Journal for Clinicians*.

[3] Lu L., Mullins C. S., Schafmayer C., Zeißig S., Linnebacher M. (2021). A global assessment of recent trends in gastrointestinal cancer and lifestyle-associated risk factors. *Cancer Communications*.

[4] Dueland S., Hagness M., Line P. D., Guren T. K., Tveit K. M., Foss A. (2015). Is liver transplantation an option in colorectal cancer patients with nonresectable liver metastases and progression on all lines of standard chemotherapy?. *Annals of Surgical Oncology*.

[5] Cleven A. H., Derks S., Draht M. X. (2014). CHFR promoter methylation indicates poor prognosis in stage II microsatellite stable colorectal cancer. *Clinical Cancer Research*.

[6] Chen P., Xi Q., Wang Q., Wei P. (2014). Downregulation of microRNA-100 correlates with tumor progression and poor prognosis in colorectal cancer. *Medical Oncology*.

[7] Jarroux J., Morillon A., Pinskaya M. (2017). History, discovery, and classification of lncRNAs. *Advances in Experimental Medicine and Biology*.

[8] Yuan J., Yue H., Zhang M. (2016). Transcriptional profiling analysis and functional prediction of long noncoding RNAs in cancer. *Oncotarget*.

[9] Tan Y. T., Lin J. F., Li T., Li J. J., Xu R. H., Ju H. Q. (2021). LncRNA-mediated posttranslational modifications and reprogramming of energy metabolism in cancer. *Cancer Communications*.

[10] Tang X., Qiao X., Chen C., Liu Y., Zhu J., Liu J. (2019). Regulation mechanism of long noncoding RNAs in colon cancer development and progression. *Yonsei Medical Journal*.

[11] Liu D., Xu B., Chen S. (2013). Long non-coding RNAs and prostate cancer. *Journal of Nanoscience and Nanotechnology*.

[12] Wang X. Y., Jian X., Sun B. Q., Ge X. S., Huang F. J., Chen Y. Q. (2020). LncRNA ROR1-AS1 promotes colon cancer cell proliferation by suppressing the expression of DUSP5/CDKN1A. *European Review for Medical and Pharmacological Sciences*.

[13] Xu G., Zhu H., Xu J. (2020). Long non-coding RNA POU6F2-AS2 promotes cell proliferation and drug resistance in colon cancer by regulating miR-377/BRD4. *Journal of Cellular and Molecular Medicine*.

[14] Wang Z. B., Zhang H. Y., Lu J. B. (2019). Expression and effects of long non-coding RNA, LINC01124, in non-small cell lung cancer. *OncoTargets and Therapy*.

[15] Li H., Yang X., Wang G. (2016). KDM4B plays an important role in mitochondrial apoptosis by upregulating HAX1 expression in colorectal cancer. *Oncotarget*.

[16] Wu G., Zhou W., Pan X. (2018). miR-100 reverses cisplatin resistance in breast cancer by suppressing HAX-1. *Cellular Physiology and Biochemistry*.

[17] Wu H., Chen J., Wang Q. (2017). Abnormal expression of HAX-1 is associated with cellular proliferation and migration in human hypopharyngeal squamous cell carcinoma. *Molecular Medicine Reports*.

[18] Huang F., Wu X., Wei M. (2020). miR-654-5p targets HAX-1 to regulate the malignancy behaviors of colorectal cancer cells. *BioMed Research International*.

[19] Jing N., Huang T., Guo H. (2018). LncRNA CASC15 promotes colon cancer cell proliferation and metastasis by regulating the miR-4310/LGR5/Wnt/*β*-catenin signaling pathway. *Molecular Medicine Reports*.

[20] Chen Y., Qiu F., Huang L. (2020). Long noncoding RNA LINC00312 regulates breast cancer progression through the miR9/CDH1 axis. *Molecular Medicine Reports*.

[21] Arnold M., Sierra M. S., Laversanne M., Soerjomataram I., Jemal A., Bray F. (2017). Global patterns and trends in colorectal cancer incidence and mortality. *Gut*.

[22] Ferlay J., Colombet M., Soerjomataram I. (2018). Cancer incidence and mortality patterns in Europe: estimates for 40 countries and 25 major cancers in 2018. *European Journal of Cancer*.

[23] Loewer S., Cabili M. N., Guttman M. (2010). Large intergenic non-coding RNA-RoR modulates reprogramming of human induced pluripotent stem cells. *Nature Genetics*.

[24] Huarte M., Guttman M., Feldser D. (2010). A large intergenic noncoding RNA induced by p53 mediates global gene repression in the p53 response. *Cell*.

[25] Mercer T. R., Dinger M. E., Mattick J. S. (2009). Long non-coding RNAs: insights into functions. *Nature Reviews Genetics*.

[26] Evans J. R., Feng F. Y., Chinnaiyan A. M. (2016). The bright side of dark matter: lncRNAs in cancer. *Journal of Clinical Investigation*.

[27] Lin C., Yang L. (2018). Long noncoding RNA in cancer: wiring signaling circuitry. *Trends in Cell Biology*.

[28] Sullenger B. A., Nair S. (2016). From the RNA world to the clinic. *Science*.

[29] Zhuang S. T., Cai Y. J., Liu H. P., Qin Y., Wen J. F. (2020). LncRNA NEAT1/miR-185-5p/IGF2 axis regulates the invasion and migration of colon cancer. *Molecular Genetics and Genomic Medicine*.

[30] Hong W., Ying H., Lin F., Ding R., Wang W., Zhang M. (2020). lncRNA LINC00460 silencing represses EMT in colon cancer through downregulation of ANXA2 via upregulating miR-433-3p. *Molecular Therapy. Nucleic Acids*.

[31] Cesana M., Cacchiarelli D., Legnini I. (2011). A long noncoding RNA controls muscle differentiation by functioning as a competing endogenous RNA. *Cell*.

[32] Bak R. O., Mikkelsen J. G. (2014). miRNA sponges: soaking up miRNAs for regulation of gene expression. *Wiley Interdisciplinary Reviews: RNA*.

[33] Dey B. K., Mueller A. C., Dutta A. (2014). Long non-coding RNAs as emerging regulators of differentiation, development, and disease. *Transcription*.

[34] Kartha R. V., Subramanian S. (2014). Competing endogenous RNAs (ceRNAs): new entrants to the intricacies of gene regulation. *Frontiers in Genetics*.

[35] Suzuki Y., Demoliere C., Kitamura D., Takeshita H., Deuschle U., Watanabe T. (1997). HAX-1, a novel intracellular protein, localized on mitochondria, directly associates with HS1, a substrate of Src family tyrosine kinases. *The Journal of Immunology*.

[36] Chao J. R., Parganas E., Boyd K., Hong C. Y., Opferman J. T., Ihle J. N. (2008). Hax1-mediated processing of HtrA2 by Parl allows survival of lymphocytes and neurons. *Nature*.

[37] Wyllie A. H., Kerr J. F. R., Macaskill I. A. M., Currie A. R., Currie A. R (1973). Adrenocortical cell deletion: the role of ACTH. *The Journal of Pathology*.

[38] Radhika V., Onesime D., Ha J. H., Dhanasekaran N. (2004). Galpha13 stimulates cell migration through cortactin-interacting protein Hax-1. *Journal of Biological Chemistry*.

[39] Vafiadaki E., Arvanitis D. A., Pagakis S. N. (2009). The anti-apoptotic protein HAX-1 interacts with SERCA2 and regulates its protein levels to promote cell survival. *Molecular Biology of the Cell*.

[40] Yedavalli V. S. R. K., Shih H. M., Chiang Y. P. (2005). Human immunodeficiency virus type 1 Vpr interacts with antiapoptotic mitochondrial protein HAX-1. *Journal of Virology*.

[41] Banerjee A., Saito K., Meyer K. (2009). Hepatitis C virus core protein and cellular protein HAX-1 promote 5-fluorouracil-mediated hepatocyte growth inhibition. *Journal of Virology*.

[42] Li X., Jiang J., Yang R. (2015). Expression of HAX-1 in colorectal cancer and its role in cancer cell growth. *Molecular Medicine Reports*.

[43] Wu Y., He R., Cai Z., Wang X., Cong L., Qiu F. *Knockdown of long noncoding RNA 01124 inhibits the malignant behaviors of colon cancer cells by regulating miR-654-5p/HAX-1*.

