# Supplementary material for: Knockdown of Long Noncoding RNA 01124 Inhibits the Malignant Behaviors of Colon Cancer Cells via miR-654-5p/HAX-1
Source: Evid Based Complement Alternat Med. 2022 Sep 20;2022:1092107. doi: 10.1155/2022/1092107 (PMC9526654; doi:10.1155/2022/1092107)

**Supplementary Table.1**

| **LINC01124_per** | **LINC01124_rc** | **mir654_per** | **mir654_rc** | **mean_per** |  |
| --- | --- | --- | --- | --- | --- |
| **sampleID** |  |  |  |  |  |
| TCGA-A6-2683-01A | 0.031444 | 17 | 6.632550 | 2955 | 3.331997 |
| TCGA-DM-A1HA-01A | 3.148121 | 1702 | 0.058357 | 26 | 1.603239 |
| TCGA-F4-6704-01A | 2.313924 | 1251 | 0.374834 | 167 | 1.344379 |
| TCGA-G4-6295-01A | 2.189997 | 1184 | 0.249141 | 111 | 1.219569 |
| TCGA-CK-4952-01A | 2.156703 | 1166 | 0.031423 | 14 | 1.094063 |
| ... | ... | ... | ... | ... | ... |
| TCGA-AA-A01T-01A | 0.005549 | 3 | 0.038157 | 17 | 0.021853 |
| TCGA-AA-3531-01A | 0.029595 | 16 | 0.013467 | 6 | 0.021531 |
| TCGA-A6-2679-01A | 0.018497 | 10 | 0.013467 | 6 | 0.015982 |
| TCGA-A6-2676-01A | 0.016647 | 9 | 0.013467 | 6 | 0.015057 |
| TCGA-AA-A00Q-01A | 0.007399 | 4 | 0.015712 | 7 | 0.011555 |

458 rows × 5 columns

Colon cancer mRNA and microRNA expression data were downloaded from the TCGA database (https://portal.gdc.cancer.gov/). Data of LINC01124(ENSG00000222033) and hsa-mir-654-5p were extracted. mRNA and microRNA data of the same samples were combined to obtain read count data of LINC01124 and hsa-mir-654-5p of 458 samples.

**Supplementary Table.2**

| **LINC01124_per** | **LINC01124_rc** | **mir654_per** | **mir654_rc** | **mean_per** | **LINC01124_rank** | **mir654_rank** |  |
| --- | --- | --- | --- | --- | --- | --- | --- |
| **sampleID** |  |  |  |  |  |  |  |
| TCGA-AA-A02Y-01A | 0.628884 | 340 | 0.017956 | 8 | 0.323420 | 154 | 1 |
| TCGA-AA-A01R-01A | 0.458716 | 248 | 0.022445 | 10 | 0.240580 | 122 | 2 |
| TCGA-AA-3556-01A | 0.580793 | 314 | 0.029179 | 13 | 0.304986 | 148 | 3 |
| TCGA-CA-5796-01A | 0.379180 | 205 | 0.031423 | 14 | 0.205302 | 107 | 4 |
| TCGA-AA-A00D-01A | 0.760210 | 411 | 0.031423 | 14 | 0.395817 | 167 | 5 |
| ... | ... | ... | ... | ... | ... | ... | ... |
| TCGA-CM-6679-01A | 0.003699 | 2 | 0.974121 | 434 | 0.488910 | 1 | 176 |
| TCGA-F4-6569-01A | 0.345886 | 187 | 0.978610 | 436 | 0.662248 | 100 | 177 |
| TCGA-5M-AAT6-01A | 0.020346 | 11 | 1.382623 | 616 | 0.701485 | 4 | 178 |
| TCGA-G4-6302-01A | 0.504957 | 273 | 1.449958 | 646 | 0.977458 | 134 | 179 |
| TCGA-A6-2683-01A | 0.031444 | 17 | 6.632550 | 2955 | 3.331997 | 8 | 180 |

180 rows × 7 columns

The read count was standardized as a percentage（read count/total count）to filter low-expression LINC01124 and hsa-mir-654-5p samples. The screening criteria was (LINC01124_per+mir654_per)/2 > 0.2 (Under this parameter, the low expression value can be removed as much as possible, and more available data can be obtained as much as possible), and 180 samples were obtained.

**Supplementary Figure 1: Knockdown of LINC01124 inhibited cell proliferation**

(A-D) CCK-8 assays were performed to assess the proliferation of HCT116 and SW480 cell lines with LINC01124-2/3. Data are presented as the mean ± SD, ***p*<0.01.


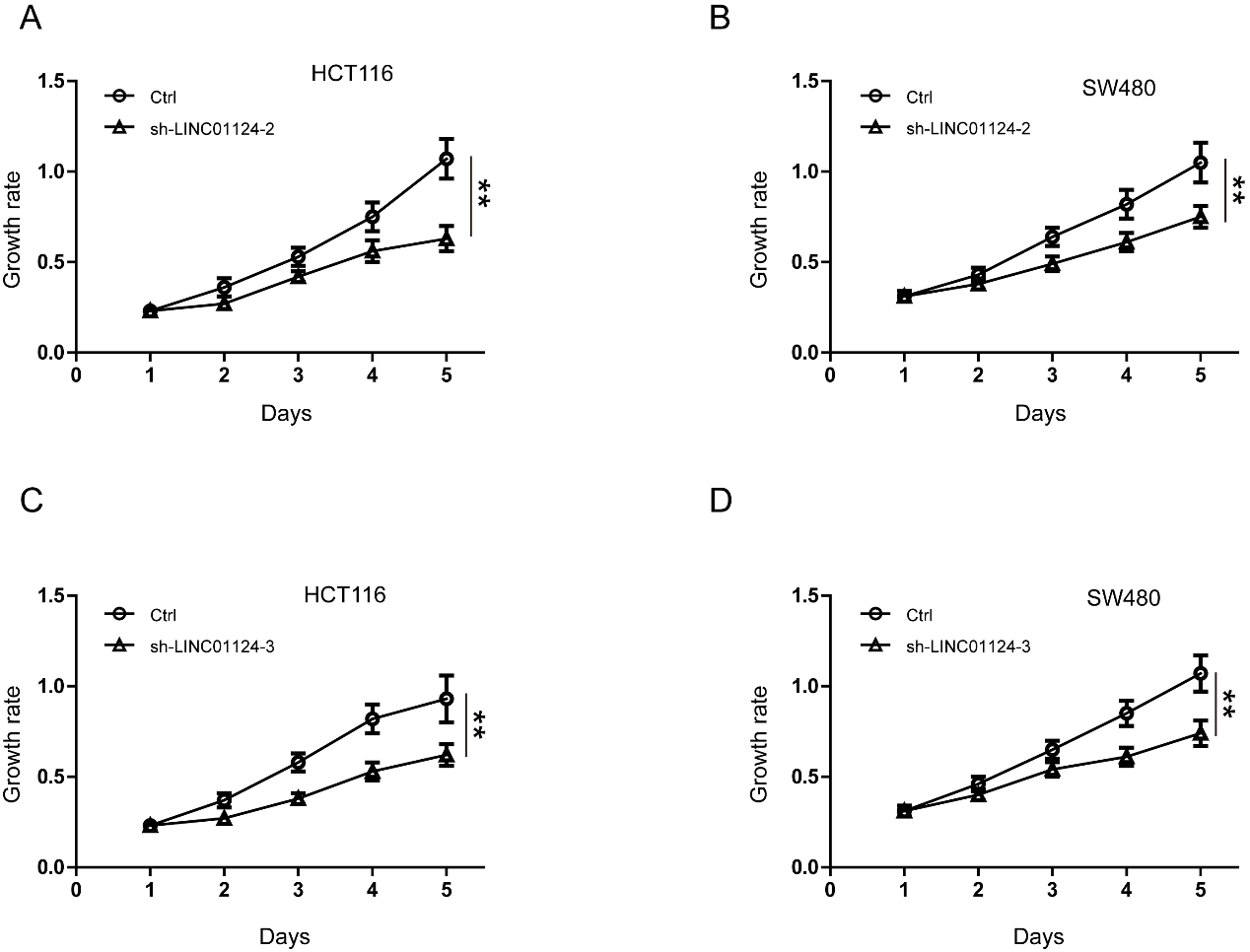


**Supplementary Figure 2: The expression of miR-654-5p in ov-LINC01124 cells**

qRT-PCR assays were performed to assess the expression of miR-564-5p in HCT116 ,SW480, LoVo and SW620 cell lines with over-expression LINC01124. Data are presented as the mean ± SD, ***p*<0.01.


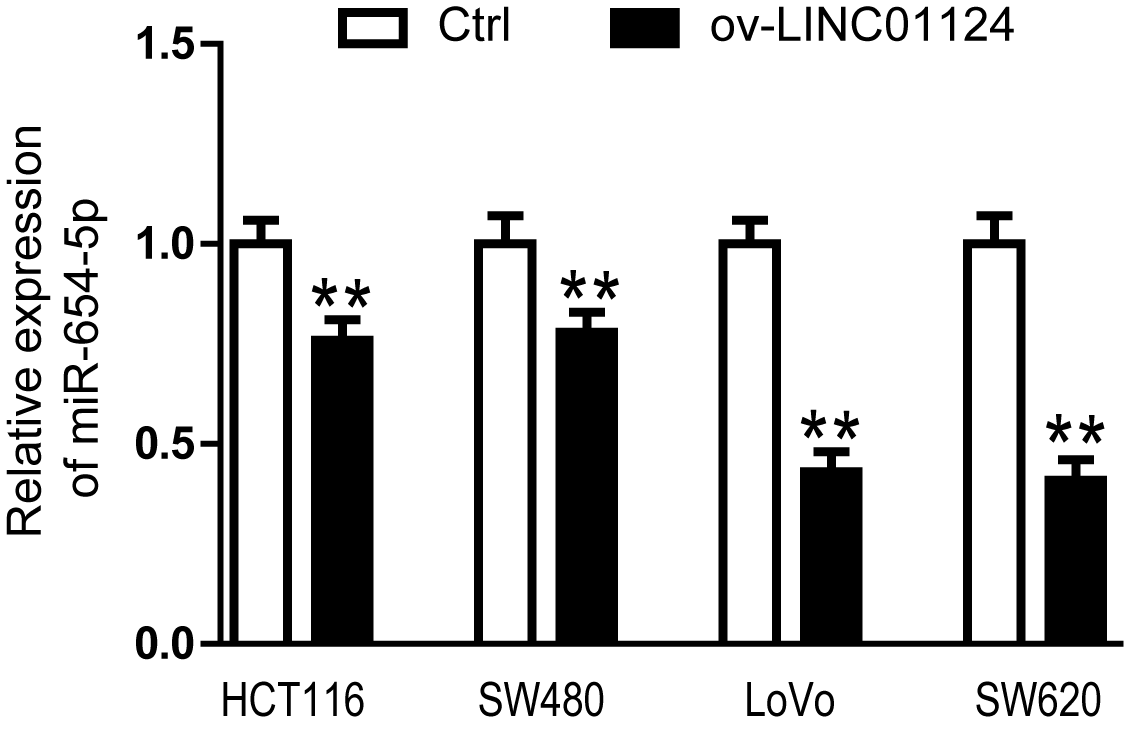


**Supplementary Figure 3: The relationship of miR-654-5p and LINC01124.**

**Analyze the relationship between miR-654-5p and LINC01124 by analyzing data from public database.**


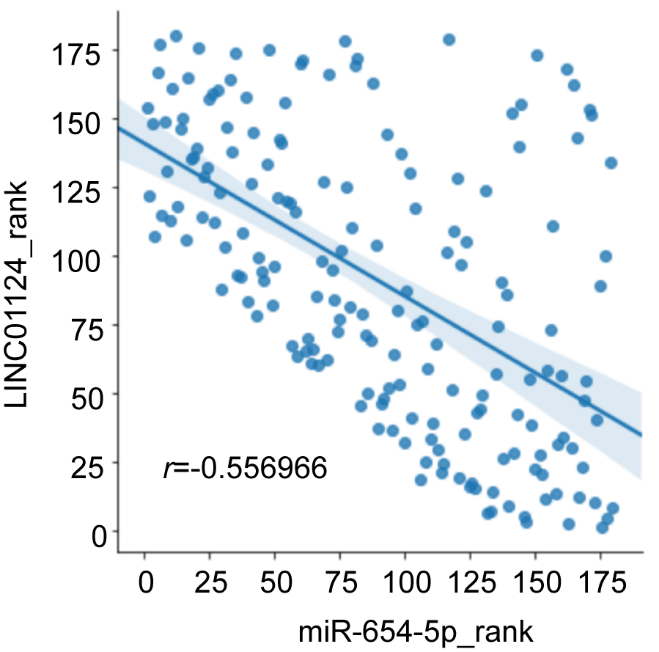

Supplement: Supplementary Materials — Table S1: analysis of the expression levels of two RNAs LINC01124 and miR-654-5p using TCGA data. Table S2: analysis of the expression levels of two RNAs LINC01124 and miR-654-5p using TCGA data. Figure S1: knockdown of LINC01124 inhibited cell proliferation. Figure S2: the expression of miR-654-5p in ov-LINC01124 cells. Figure S3: the relationship of miR-654-5p and LINC01124. [file 1092107.f1.docx]
